# Giant compressive emphysema: a rare complication of COVID-19

**DOI:** 10.1186/s12879-021-07006-6

**Published:** 2021-12-30

**Authors:** Julien Rakotoson, Johary Andriamizaka Andriamamonjisoa, Mandimbisoa Noely Oberlin Andriamihary, Solohery Jean Noël Ratsimbazafy, Roger Dominique Randrianarimalala, Rivo Andry Rakotoarivelo, Stéphane Ralandison

**Affiliations:** 1 Department of Rheumatology, Faculty of Medicine Antananarivo, Morafeno University Hospital Toamasina, 501 Toamasina, Madagascar; 2 Department of Infectious Diseases, Faculty of Medicine Antananarivo, Joseph Raseta Befelatanana University Hospital Antananarivo, 101 Antananarivo, Madagascar; 3 Department of Internal Medicine, Faculty of Medicine Antananarivo, Military Hospital Soavinandriana Antananarivo, 101 Antananarivo, Madagascar; 4 Department of Infectious Diseases, Faculty of Medicine Andrainjato Fianarantsoa, University Hospital Tambohobe Fianarantsoa, 301 Fianarantsoa, Madagascar; 5 Department of Cardiology, Faculty of Medicine of Antananarivo, Joseph Rasta Befelatanana University Hospital, 101 Antananarivo, Madagascar

**Keywords:** Emphysema, Giant, Coronavirus disease 2019, CT scan

## Abstract

**Background:**

The severe acute respiratory syndrome coronavirus-2 (SARS-CoV-2) is a new ribonucleic acid (RNA) beta-coronavirus, responsible for a worldwide pandemic. Very few cases of SARS-COV-2-related emphysema have been described, except among patients with chronic obstructive pulmonary disease. The thoracic CT scan is the key examination for the diagnosis and allows to evaluate the severity of the pulmonary involvement. The prognosis of the patient with giant emphysema (GE) on coronavirus disease 2019 (COVID-19) in critical or severe form remains poor. We report an original case of COVID-19 pneumonia, critical form, complicated by a giant compressive left emphysema of 22.4 cm in a young subject without respiratory comorbidities.

**Case presentation:**

A 34-year-old man was hospitalized for left laterothoracic pain. He had no prior medical history. The physical examination revealed tympany on percussion of the left lung. The CT scan confirmed COVID-19 pneumonia with 95% lung involvement**.** Also, the presence of a voluminous left sub pleural emphysema of 22.4 cm with compression of the ipsilateral pulmonary parenchyma as well as the mediastinal structures towards the right side**.** The diagnosis COVID-19 pneumonia, critical form, complicated by a compressive left giant emphysema was made. He was put on oxygen, a dual antibiotic therapy, a corticotherapy, and curative doses of enoxaparin. A thoracic drainage surgery was performed at 24th day of hospitalization, which confirmed the giant emphysema. The patient remains on long-term oxygen therapy.

**Conclusion:**

The COVID-19 has polymorphic manifestations, pneumonia is the most important one. There are relatively few reports associating COVID-19 and emphysema; furthermore, reports associating COVID-19 and giant emphysema are extremely scarce. CT scans can confirm the diagnosis and differentiate it from a pneumothorax. The pulmonary prognosis of the association of COVID-19 in its severe or critical form with giant emphysema remains poor.

## Background

The severe acute respiratory syndrome coronavirus-2 (SARS-CoV-2) is a novel single-stranded ribonucleic acid (RNA) beta-coronavirus, which emerged in China in December 2019, responsible for the coronavirus disease 2019 (COVID-19), which is causing the current global pandemic [1]. Despite the polymorphism of this disease, pulmonary involvement remains the predominant presentation causing hypoxemic lung disease [2, 3]. Emphysema is the consequence of an irreversible destruction of the pulmonary parenchyma, with damage to the respiratory bronchiole and alveolar walls, causing an enlargement of the airways and responsible for chronic respiratory insufficiency. Giant emphysema (GE), on the other hand, causes acute respiratory failure and a severe compressive phenomenon. Since the pandemic, very few cases of SARS-COV-2 related emphysema have been described, except among patients with chronic obstructive pulmonary disease (COPD) [2, 3]. Our aim is to report an original case of GE on SARS-CoV-2 lung disease, in a young subject without comorbidity.

## Case presentation

A 34-year-old man was hospitalized in Antananarivo-Madagascar for left laterothoracic pain associated with dyspnea. He reported no cigarette nor alcohol consumption, and denied any prior medical history including diabetes, hypertension, and respiratory diseases. He has no known respiratory history. There was no family history of alpha-1 antitrypsin deficiency, no notion of pneumothorax or spontaneous emphysema. He had been presenting for 10 days with headaches, diffuse arthromyalgia, and a hacking cough in a febrile context. Two days before his admission, the patient suffered from significant asthenia, followed by progressive dyspnea, chest pain aggravated by coughing and change of position.

The physical examination revealed pulsed oxygen saturation (SpO2) of 77%, polypnea of 35 cycles per minute, blood pressure of 160/80 mmHg, heart rate of 117 beats per minute, temperature of 36.7 °C, and body mass index (BMI) of 23.1 kg/m^2^. The patient was obnubilated, had difficulty speaking, with signs of acute respiratory distress, condensation syndrome of the entire right lung, auscultatory silence and tympany on percussion of the left lung. There was no subcutaneous snowy crepitus of the thoracic region. There was right deviation of regular heart sounds, turgidity of the jugular veins, without edema of the lower extremities or ascites.

**Fig. 1:**
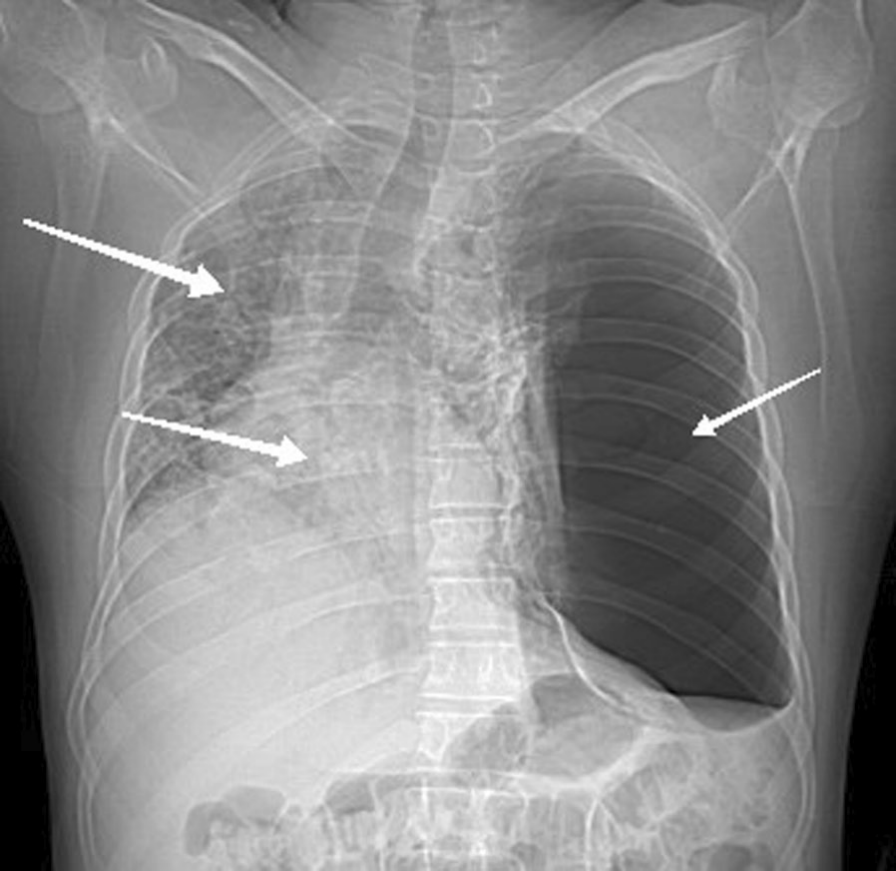
Chest CT scan without contrast injection: the chest X-ray at the patient's bed showed an alveolar syndrome of the right lung (arrow: right/up) with right deviation of the mediastinum (arrow: right/down) and clarity of the right lung except at the apex (arrow: left)

Lab analyses made in the 2nd day of hospitalization showed a hyperleukocytosis of 27 × 10^3^ k/µL, including 96% (25 × 10^3^ k/µL) neutrophils, 2% (0.54 × 10^3^ k/µL) lymphocytes, a discrete thrombocytosis of 475 × 10^3^ k/µL, and a C-reactive protein of 75 mg/L. Renal and hepatic tests were unremarkable. D-dimer was elevated to 2571 ng/mL. The nasopharyngeal COVID-19 polymerase chain reaction (PCR) performed on the 3rd day of hospitalization was positive. Human immunodeficiency virus (HIV) serology was negative. The chest X-ray at the patient's bed on the 2nd day of hospitalization showed an alveolar syndrome of the right lung with right deviation of the mediastinum and clarity of the right lung except at the apex (Fig. 1).

The diagnosis of COVID-19 was made, with a doubt on the complication: left emphysema or left pneumothorax. He was put on oxygen at 20 L/min through a high-flow nasal cannula connected to an oxygen cylinder; a dual antibiotic therapy with ceftriaxone slow direct intravenous 1 g daily for 10 days, associated with a roxythromycin 150 mg tablet, twice daily for 10 days; a corticotherapy with dexamethasone intravenous direct: 24 mg daily for 3 days, then 12 mg daily for 7 days, then 8 mg daily for 7 days, then 4 mg daily for 6 day. A potassium supplementation with 600 mg × 3 dose per day for 22 days; and curative doses of enoxaparin subcutaneous 0.6 ml every 12 h for 10 days. The evolution was favorable with disappearance of signs of respiratory distress, with a SpO2 of 92% under 8 L of oxygen at 14th day of hospitalization.

**Fig. 2:**
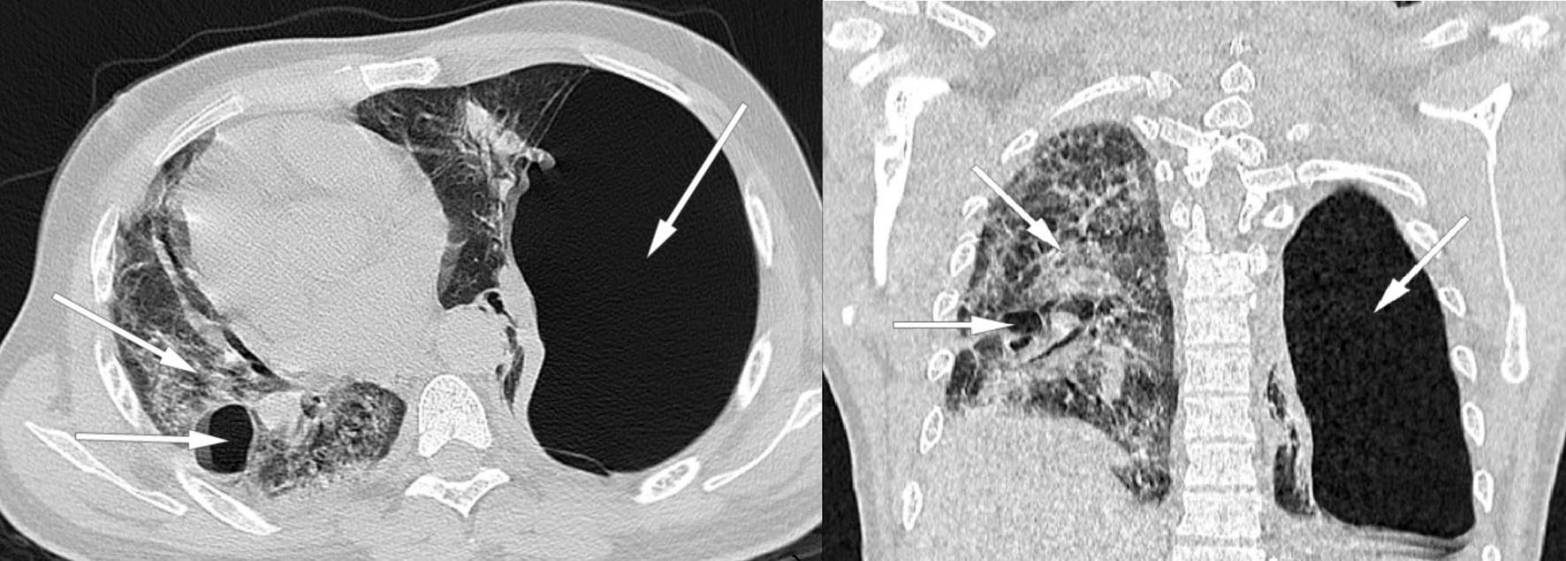
(Coronal and axial section): ground glass opacities with multilobar and multisegmental internal reticulations with pulmonary involvement about 95% suggestive of SARS-Cov-2 infection (arrow: right/up). Voluminous left sub pleural emphysema of 22.4 cm and some pulmonary emphysema bullae on the right (arrow: left. Fig. 3, arrow: left). Compression of the ipsilateral lung parenchyma and deviation of the mediastinal structures to the right side by a compressive phenomenon are noted

**Fig. 3:**
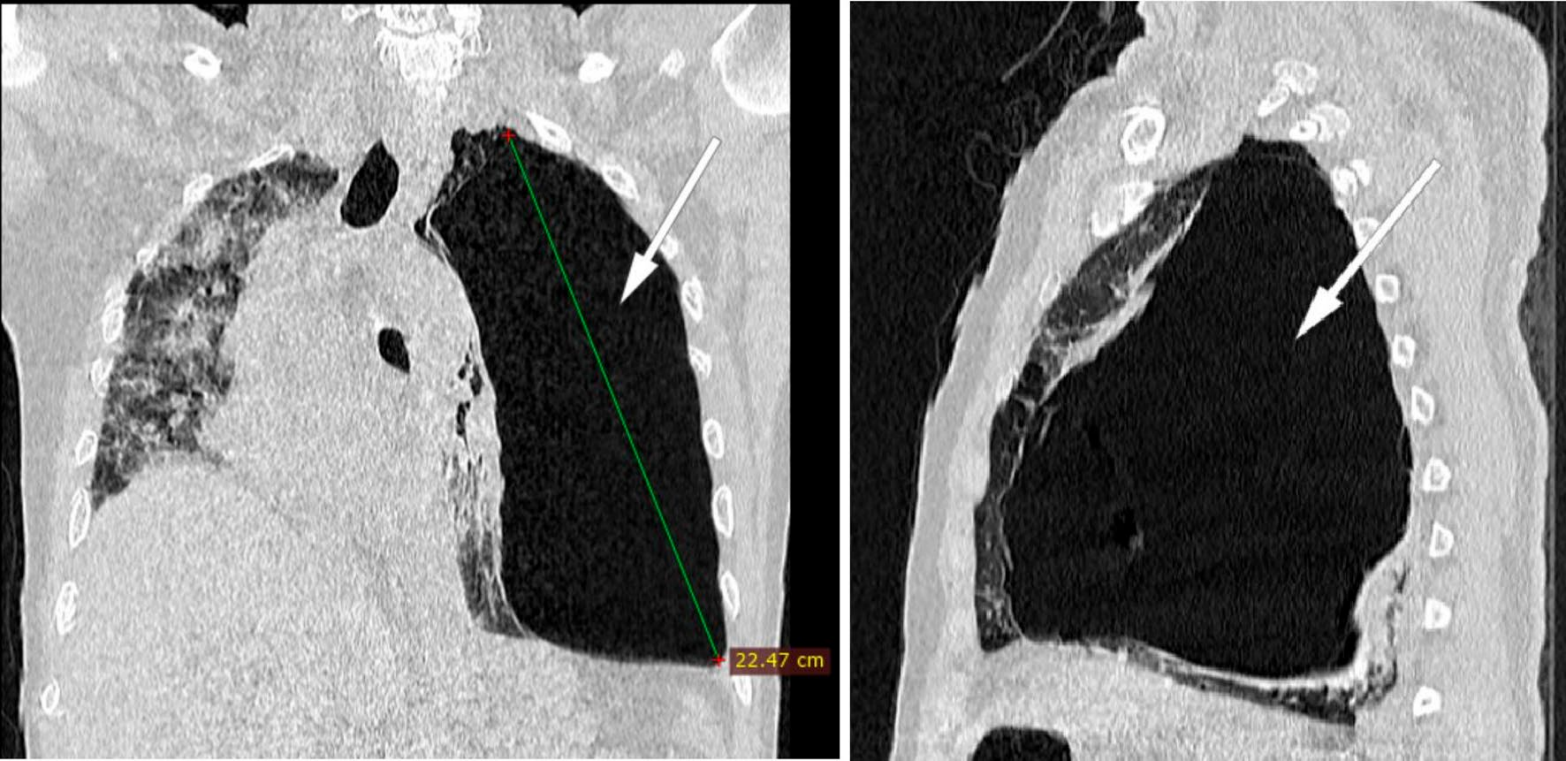
(Coronal and sagittal section): ground glass opacities with multilobar and multisegmental internal reticulations with pulmonary involvement about 95% suggestive of SARS-Cov-2 infection (Fig. 2, arrow: right/up). Voluminous left sub pleural emphysema of 22.4 cm and some pulmonary emphysema bullae on the right (Fig. 2, arrow: left. arrow: left). Compression of the ipsilateral lung parenchyma and deviation of the mediastinal structures to the right side by a compressive phenomenon are noted

The thoracic scanner was done late at 15th day of hospitalization because of the dependence on oxygen therapy and the impossibility of moving the patient with a worsening of desaturation at the least movement. In addition, the radiology center is not on site, requiring a trip with an ambulance that has a capacity of 10 L of portable oxygen. The injection of contrast medium is not done because of the patient's lack of money. The result of the CT scan is in favor of a viral pneumonia on SARS-CoV-2 with pulmonary involvement of about 95% of the parenchyma, by the presence of ground glass opacities with multilobar and multisegmental internal reticulations**.** Also, the presence of a voluminous left sub pleural emphysema of 22.4 cm with compression of the ipsilateral pulmonary parenchyma as well as the mediastinal structures towards the right side**.** At the same time, we note the presence of some pulmonary emphysema bullae on the right side **(**Figs. 2, 3).

The diagnosis of COVID-19 pneumonia, critical form, complicated by a compressive left GE was made. Pneumothorax was ruled out by the presence of lung parenchyma at the apex and the left pulmonary base. Also, the absence of retraction of the left lung on the pulmonary hilum.

However, the tympanism of the left lung thorax and the deviation of the heart sounds persisted. On the 22nd day of hospitalization: the patient remained oxygenorequerent with SpO2 at 94% under 2 L of oxygen and desaturation at the slightest effort. A thoracic surgery was performed at 24th day of hospitalization, which confirmed the GE. The Surgery was done under general anesthesia with mechanical ventilation by a thoracotomy, then a bullectomy followed by placement of a chest tube. The chest tube was removed on day 6 of the operation. The operation was successfully performed to overcome the acute respiratory status. Nevertheless, the patient remains on long-term oxygen therapy by nasal cannula. The patient survived 2 months after his thoracic drainage, following a recurrence severe acute pneumonia.

## Discussion and conclusions

In the absence of pathological lungs, COVID-19 can cause giant emphysema. The severity is related to the size of the emphysema, which is a source of compressive phenomenon. The CT scan is the key examination for diagnostic confirmation. The prognosis of the association remains poor.

Emphysema is a lung disease, characterized by the irreversible destruction of the respiratory bronchiole and the alveolar wall that lines the lungs. This leads to an enlargement of the distal airways with the formation of intra-parenchymal air bubbles [4]. It is purely related to COVID-19 if an infected patient has no history of chronic obstructive pulmonary disease or a history of familial alpha-1 antitrypsin deficiency. The association of emphysema with COVID-19 was less described in the first wave in 2020, but seems to be more numerous in the 2nd wave of 2021. Our case finds its originality in relation to the size of the emphysema, voluminous, measuring 22.4 cm long axis and its acquired character related to COVID-19. Also, because of the young age of our patient, non-smoker, which can eliminate the aggravation of pre-existing emphysema or blebs ruptures.

The mechanism of our patient's GE may be different from that of Sun et al. Our patient had the signs of GE on admission (tympany with respiratory distress, and the image of emphysema in the chest X-ray). In other words, emphysema symptoms were the main reason for our patient's consultation. On the other hand, the case reported by Sun et al. presented the signs of emphysema during hospitalization where the patient had already received oxygen therapy at 20 L/min [5]. COVID-19 itself and its treatment by excessive oxygen flow increase during desaturation could explain the occurrence of emphysema» [6]. The pathogenesis of emphysema primarily involves an inflammatory process in the lungs during various infections. Macrophages (CD 68+), neutrophils and CD8 T cells act on resident cells such as epithelial cells and fibroblasts via mediators (interleukin 8, TNF alpha, leukotriene) of inflammation, destroying the lung parenchyma. This mechanism occurs in the inflammatory phase of COVID-19 with alveolar-interstitial lung disease, in addition to the oxidative stress (formation of free radicals) created by the inflammatory process (2nd mechanism). The 3rd mechanism is an imbalance between pulmonary proteases–antiproteases often of genetic origin. The alpha 1 antitrypsin protein (protease inhibitor) plays a protective role. In case of its deficiency, this role is no longer ensured and the progressive destruction of the pre-existing alveolar walls causes the occurrence of emphysema [7]. This hypothesis is less likely in our case because there is no history of spontaneous pneumothorax in the family.

Any source of increased intra-thoracic pressure (exertion, coughing, constipation …) can aggravate the size of an underlying emphysema or cause the rupture of a pre-existing bleb. Excessive ventilation can also aggravate or cause emphysema. Our patient has presented pulmonary tympany as soon as they admitted and had no invasive ventilation before the thoracic scanner. This eliminates the contribution of ventilation to the development of emphysema in this patient.

Critical CT involvement in COVID-19 is defined as 75% or more lung involvement. Our case presented up to 95% lung involvement, aggravated by the presence of a GE of 22.4 cm. This association increases the risk of mortality of the disease [7, 8].

The CT scan is ideal imaging method for the diagnosis of emphysema and for the evaluation of its impact. It can differentiate between emphysema and pneumothorax or blebs. In case of COVID-19, chest CT has a sensitivity of 91.9% for the diagnosis, considered as a reference examination even in the early stage of the infection [9, 10]. It is therefore essential to perform a chest CT scan for any severe or critical form of COVID-19, to assess the severity of the lung involvement, to look for associated lesions, to have an idea of the prognosis and to provide a means of follow-up. Most COVID-19 related emphysemas described in the literature are small, with possible association with pneumomediastinum and pneumothorax. Regarding the therapeutic management, our patient was receiving oxygen therapy and surgery. The emphysema described in the literature was treated with oxygen therapy only [5]: non-invasive ventilation, high Flow Nasal Cannula and invasive mechanical ventilation [6]. In our case, surgery was necessary in view of the presence of signs of poor tolerance of the GE.

The association of COVID-19 and pulmonary emphysema is less described in the literature. Even more, GE is exceptional. It should be evoked in front of the asymmetry of the pulmonary examination with tympanism and the presence of signs of acute right heart failure. Only the CT scan can confirm the diagnosis and differentiate it from a pneumothorax. The pulmonary prognosis of the association of COVID-19 in its severe or critical form with GE remains poor (Table 1).

**Table 1:** Summarizing the clinical and laboratory characteristics of patient

Chronology of the event	Clinical and biological characteristics of the patient
10th day before his hospitalization	Influenza-like syndrome, febrile context
2nd day before his hospitalization	Progressively worsening dyspnea and chest pain that are accentuated by coughing and changing position
On admission	Clinical characteristics: No sign of shock Acute respiratory distress syndrome with pulse oxygen saturation 77% on room air, polypnea at 35 cycles per minute Condensation syndrome of the entire right lung Tympany and auscultatory silence of the left lung
On admission	Biological characteristics: Hyperleukocytosis: 27 × 10^3^ k/µL, 96% (25 × 10^3^ k/µL) neutrophils, 2% (0.54 × 10^3^ k/µL) lymphocytes Thrombocytosis: 475 × 10^3^ k/UL C-reactive protein: 75 mg/L D-dimer: 2571 ng/mL The nasopharyngeal COVID-19 polymerase chain reaction (PCR): positive HIV serology: negative
22nd day of hospitalization	Disappearance of signs of respiratory distress Pulse oxygen saturation 94% at 2L/min oxygen Desaturation at the slightest effort
24th day of hospitalization	Bullectomy surgery, with thoracic drainage
From the 7th day post-op	Long-term oxygen-dependent, at home
2 months after surgery	Died of a recurrence severe acute pneumonia

## Data Availability

We cannot put the scanner online with the patient's information because of the confidentiality rule. On the other hand, imaging with patient information is available at the first author.

## Abbreviations

BMI: Body mass index
COPD: Chronic obstructive pulmonary disease
COVID-19: Coronavirus disease 2019
CT: Computerized tomography
GE: Giant emphysema
HIV: Human immunodeficiency virus
RNA: Ribonucleic acid
SARS-CoV-2: Severe acute respiratory syndrome coronavirus-2
SpO2: Pulsed oxygen saturation
